# Trait anxiety does not correlate with metacognitive confidence or reminder usage in a delayed intentions task

**DOI:** 10.1177/1747021820970156

**Published:** 2020-11-12

**Authors:** Peter A Kirk, Oliver J Robinson, Sam J Gilbert

**Affiliations:** 1 Institute of Cognitive Neuroscience, University College London, London, UK; 2 Department of Experimental Psychology, University College London, London, UK

**Keywords:** Memory, metacognition, anxiety

## Abstract

Setting external reminders provides a convenient way to reduce cognitive demand and ensure accurate retrieval of information for prospective tasks. Recent experimental evidence has demonstrated that the decision to offload cognitive information to external resources is guided by metacognitive belief, that is, individuals’ confidence in their unaided ability. Other work has also suggested a relationship between metacognitive belief and trait anxiety. In the present study (*N* = 300), we bridged these two areas by investigating whether trait anxiety correlated with metacognitive belief and—consequently—propensity to offload information in a delayed intentions paradigm. Participants received a financial reward based on their ability to remember targets. However, participants could take a reduced reward per target if they decided to use reminders. We replicated previous findings that participants were biased to use more reminders than would be optimal, and this bias was correlated with metacognitive judgements. However, we show no evidence that trait anxiety held a relationship with metacognitive belief or reminder usage. Indeed, Bayesian analyses strongly favoured the null. Therefore, variation in self-reported trait anxiety does not necessarily influence confidence and strategy when participants remember delayed intentions.

## Introduction

As working memory capacity is constrained (Cowan, 2010), it is common for humans to offload relevant information onto the external environment for prospective tasks (Gilbert, 2015). This is an example of “cognitive offloading” (Finley et al., 2018; Risko & Gilbert, 2016). For example, during a lecture, you may note down questions to ask at the end, or you may create an entry in your calendar to remember your friend’s birthday. However, offloading behaviours vary greatly, and it is intrinsically linked to the way many interact daily with their physical environment (e.g., leaving keys by the door) and technological resources (e.g., smartphone alarms). The use of reminders reduces cognitive demand and improves memory task performance (Gilbert, 2015; Hu et al., 2019; Risko & Dunn, 2015).

Recent studies have begun to investigate how individuals decide whether or not to use reminders. Evidence suggests that individual differences in cognitive confidence may guide one’s decision to offload information (Boldt & Gilbert, 2019). Moreover, the accuracy of individuals’ judgement of their own abilities (metacognitive bias) is related to how optimal these decisions are (see Gilbert et al., 2020). For instance, individuals who are underconfident in their ability will tend to set reminders for delayed intentions which they would have remembered anyway using their own memory. However, metacognitive error cannot explain suboptimal reminder-setting in full. For example, excessive reminder-setting can be observed both in the presence of under- and overconfidence (see Gilbert et al., 2020, Experiment 3). Thus, although confidence may guide one’s decisions to set reminders, there remains a gap in the literature as to what other factors contribute to individuals’ tendency to offload delayed intentions. An unexplored avenue is whether affective processes may influence these decisions.

Anxiety is one emotional experience known for its influence on non-affective cognition. There are a myriad of studies providing evidence that anxiety affects many cognitive domains, manifesting in altered behaviours (for a review, see Robinson et al., 2013). In tasks requiring memory for delayed intentions, a mixed picture has emerged, with some studies finding a relationship between anxiety and task performance (Arnold et al., 2015; Kliegel & Jäger, 2006), but not others (Cuttler & Graf, 2008). However, irrespective of memory performance, no previous study has tested the relationship between anxiety and propensity to set reminders in an experimental task. Prior research has established a link between anxiety and metacognitive belief, such that individuals experiencing higher anxiety often have lower cognitive confidence (Spada et al., 2010; Wells & Cartwright-Hatton, 2004). In the context of delayed intentions, it would then be expected that increased anxiety might increase propensity to use reminders.

The correlations observed between metacognition and anxiety largely rely on the Metacognitions Questionnaire 30 (MCQ-30; Wells & Cartwright-Hatton, 2004), which includes asking participants to self-rate how misleading and poor their memory is. Although this may be a clinically useful construct (Meyer et al., 1990), this non-specific self-report does not necessarily relate to actual memory ability. An alternative approach is to ask participants to judge their performance level on a specific task, based on a measure such as percent accuracy (e.g., Gilbert et al., 2020). This can then be compared against the actual accuracy level to yield a measure of metacognitive bias, that is, the difference between participants’ subjective rating of accuracy and their actual performance level. To our knowledge, no study has linked global measures of anxiety to task-specific metacognitive bias in a memory task. Consequently, there is no indication as to whether anxious individuals’ lowered confidence is related to actual memory ability and/or whether they engage in suboptimal reminder-setting behaviours. Given the frequency at which we assess our internal memory abilities, and consequently rely on offloading behaviours, significant underconfidence could potentially lead to unhealthy oversetting of reminders. Being aware of maladaptive cognitions and behaviour is in fact central to the first-line psychological treatment for anxiety: cognitive-behavioural therapy (Kovacs & Beck, 1978). Thus, delineating whether anxiety results in metacognitive bias and oversetting of reminders presents directly translatable information for clinicians. For example, this could provide insight as to whether clinicians should provide additional metacognitive assessments and interventions in the domain of prospective memory for patients with anxiety disorders (Wells, 2009).

Although the literature implies a possible indirect relationship between anxiety and reminder usage mediated via metacognition, this does not rule out that anxiety could influence reminder-setting in other ways. In addition to affecting cognitive confidence, anxiety might more directly influence reminder-setting. Some have proposed that anxiety may be detrimental to behaviour as worrying thoughts (a primary component of anxiety) use up memory resources, and thus capacity (Eysenck et al., 2007; Seibert & Ellis, 1991), suggesting a potentially greater need for reminders. Recent research has demonstrated however that the increased cognitive load caused by anxiety does not always explain effects on cognition (e.g., time perception, see Sarigiannidis et al., 2020). Attentional control theory (Eysenck et al., 2007) also suggests that anxiety influences behaviour by shifting attentional resources in search of threatening stimuli, reducing attention to the present task (unless it involves threatening stimuli). Irrespective of dimensional underpinnings, no prior research has yet established whether a correlation exists between anxiety and offloading behaviour. We therefore conducted a preregistered (https://osf.io/zguhj/) study to explore the relationship between anxiety, metacognition, and reminder-setting.

Our experimental task required participants to remember delayed intentions over a brief time period. This could be considered to involve “prospective memory,” an umbrella term referring to situations where individuals must remember to perform actions in the future (Brandimonte et al., 1996; Kliegel et al., 2008; Scullin et al., 2015). However, some authors have argued that the term prospective memory is more appropriate for memory tasks involving a longer retention interval (see Graf & Uttl, 2001, for discussion). We note that a task similar to the present one has previously been shown to predict participants’ prospective memory performance over longer retention intervals of up to a week (Gilbert, 2015). Nevertheless, to avoid confusion, we use the more theoretically neutral phrase “delayed intentions task” rather than “prospective memory task” below.

### Hypotheses

The main aim of the present study was to test the following six preregistered hypotheses in relation to a delayed intentions task:

1. Trait anxiety will negatively correlate with participants’ accuracy.
2. Trait anxiety will negatively correlate with participants’ confidence.
3. Trait anxiety will negatively correlate with participants’ metacognitive bias (trait anxiety results in greater underconfidence or less overconfidence).
4. Trait anxiety will positively correlate with participants’ propensity to use reminders.
5. Trait anxiety will positively correlate with participants’ bias towards the use of reminders (trait anxiety results in increased overuse of reminders or reduced underuse of reminders).
6. Both anxiety and metacognitive bias will account for unique variance in a model of reminder bias.

In sum, this study aimed to assess the link between anxiety and metacognitive bias in memory for delayed intentions.

## Method

### Preregistration

All hypotheses, experimental methods, and planned analyses were preregistered prior to data collection. Materials and source code are uploaded on the Open Science Foundation (https://osf.io/zguhj/). We note the following deviations from our planned analyses:

- We originally planned to conduct frequentist tests for all our analyses. However, we supplemented these with Bayesian equivalents to aid in the interpretation of our findings.
- We have provided post hoc, supplementary analyses which help address concerns such as counterbalancing, screening procedure, and exclusion criteria (Supplementary Materials 5–9).

### Participants

Participants were recruited from the Amazon Mechanical Turk website (http://www.mturk.com), an online marketplace in which participants receive payment for completion of web-based tasks (Crump et al., 2013). Ethical approval was received from the UCL Research Ethics Committee (1584/003) and participants provided informed consent before participating in the study.

A statistical power analysis was performed with G*Power 3.1 for sample size estimation. No prior research has directly investigated the role of affective systems on reminder-setting. We therefore decided to power our study based on our previous research on metacognitive bias and offloading (Gilbert et al., 2020, Experiment 2, metacognitive bias-reminder bias correlation [unadvised group]), which found a correlation of *r* = –.31. We chose a sample size of 300, which would give us sufficient power to detect a ~50% reduction of this effect (*r* = .161, α = .05, 1 – β = .8). As in earlier studies (Gilbert, 2015), participation was restricted to volunteers aged at least 18 years, located in the United States. Participants who had already taken part in the present study were blocked to ensure a fresh sample of participants. We also restricted inclusion to participants with a minimum of 90% Mechanical Turk approval rate. Participation took approximately 45 min, for which participants received a base payment of $2, plus an additional bonus payment of up to $8.67 (see below). Our final sample (*N* = 300) had a mean age of 37.81 years (*SD* = 10.97; minimum = 21; maximum = 72); 190 reported their gender as male, 108 as female, and 2 as other.

### Design

#### Anxiety/worry measures

Trait anxiety was measured using the trait section of the State Trait Anxiety Inventory (STAI; Spielberger, 1983), a 20-item questionnaire which provided us a global measure of individual differences in anxiety. This was the key measure of anxiety. We selected the trait section as this is a temporally stable attribute previously shown to correlate with cognitive confidence (Wells & Cartwright-Hatton, 2004). However, we also report that the trait section appears to already correlate very highly with the state section of the questionnaire, *r*(1058) = .83, *p* < .0001, BF_10_ > 1,000; (Supplementary Material 7. As an additional measure for exploratory follow-up analyses, we also included the penn state worry questionnaire (PSQW) (Meyer et al., 1990), a 16-item questionnaire which provided us a metric of individual differences in worrying thoughts.

#### Offloading task

We used a modified version of a previously verified delayed intentions task (Gilbert et al., 2020). Participants chose between remembering intentions using their own memory (in which case they earned full reward for each remembered item) or using external reminders (in which case they earned a smaller reward, which varied from trial to trial). This allowed us to examine not only participants’ propensity to use reminders but also the optimality of their reminder-setting strategy. For example, suppose a participant can achieve 55% accuracy using their own memory and 100% accuracy using reminders. Given a choice between 10 points per item (using own memory) or 5 points per item (using reminders), the optimal strategy is to use one’s own memory. However, if offered 6 points per item using reminders, it is optimal to use reminders. In this way, we could compare participants’ reminder-setting strategy with the optimal strategy, to investigate the extent to which they were biased towards using external reminders versus their own memory.

**Figure 1.**
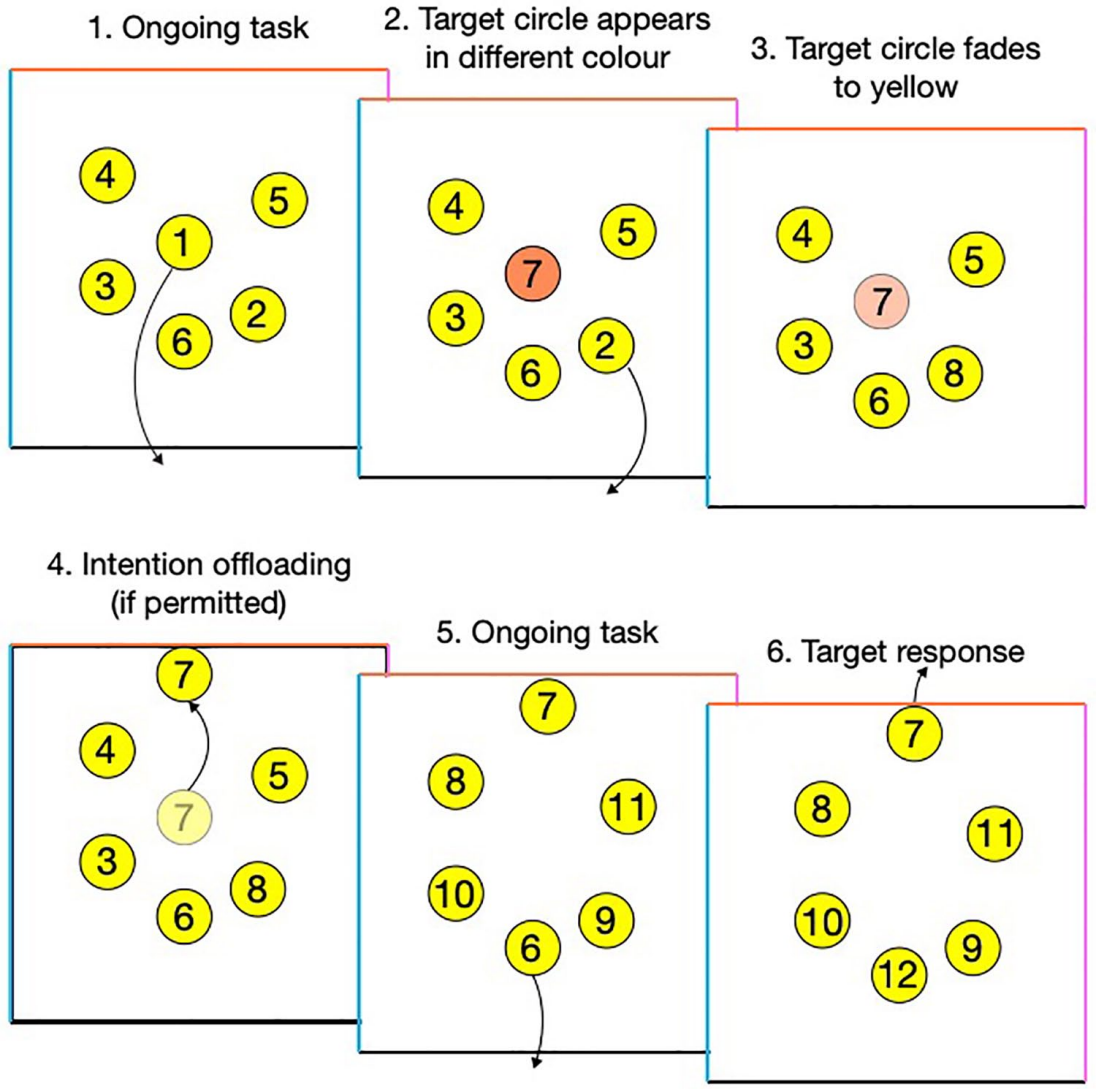
Schematic of an example trial: (1) Participants dragged “”circles in sequential order to the bottom edge of a box. Every time a circle was removed from the box, it was replaced with a new one; (2) sometimes, new target circles appeared in a different colour, signifying a delayed intention to later drag the target to a coloured edge; (3) the colour faded after 2 s. (4) participants were sometimes permitted to set reminders by immediately dragging target circles to the instructed edge when they appeared; (5) participants continued to drag the next circles in the sequence to the bottom of the box; and (6) after dragging the appropriate circles in the sequence, they could then execute the delayed intention to drag the target to circle to the correct edge.

During each trial, participants used their computer mouse to drag 25 numbered circles in sequential order (1–25) to the bottom edge of a box (Figure 1). Six yellow circles were shown on the screen at once, and each time a circle was removed from the box, it was replaced with a new one (e.g., after dragging “1” to the bottom, a new circle labelled “7” appeared in its place). Sometimes, new circles initially appeared in blue, orange, or purple, which corresponded to the colours of the left, top, and right edges of the box (which was displayed throughout all trials). These circles then faded to yellow after 2 s. This constituted an instruction for a delayed intention to drag these “target” circles to a different edge of the box. For instance, if a target circle (e.g., 7) initially appeared as orange, participants needed to remember this instruction while they dragged circles 2 to 6 to the bottom of the box (by which time the target circle had faded to yellow). When they finished this, they could then execute the intention to drag 7 to the top. Across an entire trial of 25 circles, 10 target circles were presented. This meant that participants had to encode multiple intentions and were unlikely to remember all of them if they relied on their internal memory ability. Alternatively, if participants set reminders, they could offload the intentions by immediately dragging target circles to the instructed edge when they appeared (e.g., dragging an orange 7 next to the top edge of the box; this could be done immediately upon its presentation rather than having to wait for it to fade to yellow first). Its location then acted as a reminder when the participant reached this number in the sequence, analogous to leaving an object by the front door so that you remember it when leaving the house tomorrow.

**Figure 2.**
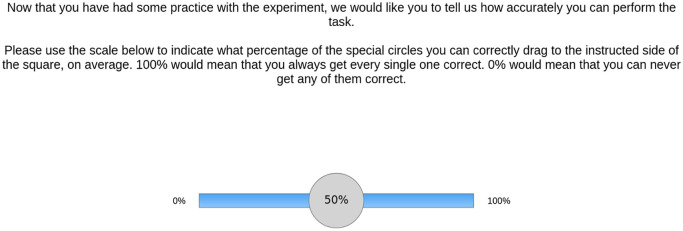
Metacognitive confidence rating screen. After completing a series of practice trials, participants rated what percentage of target circles they believed they could accurately remember. These judgements were collected once at the beginning of the experiment, and separately for the internal and external strategies. For the confidence measure following the forced-external practice, participants were instead prompted with “Now that you have practiced doing the task using reminders, we would like you to tell us how accurately you can perform the task when you use this strategy.” This provided us with our metacognitive confidence measure.

The main experimental paradigm alternated between different trial types. On some trials, participants were forced to either use their own memory or external reminders. We refer to these as forced internal and forced external, respectively. On other trials, participants were given a choice between using their own memory and earning 10 points per remembered item or using reminders and earning a smaller number of points between 2 and 8 (free choice). This allowed us to calculate the optimal strategy (based on accuracy on the forced-internal/external trials) and compare this against actual reminder-setting strategy (based on the free-choice trials). Prior to starting the main experiment, participants were instructed and practised the task with and without target circles (forced internal). They were not allowed to continue until they completed these practice trials successfully. Subsequently, they practised again on the forced-internal trial type, after which they provided a measure of how confident they were at their ability to perform the task (Figure 2). Following this, they were made aware of the ability to use reminders in the task. They then practised again, but on the forced-external trial type, after which they provided a measure of how confident they were at their ability to perform the task using reminders. This enabled us to investigate whether participants’ reminder-setting strategy was related to their metacognitive beliefs about their ability to perform the task.

**Figure 3.**
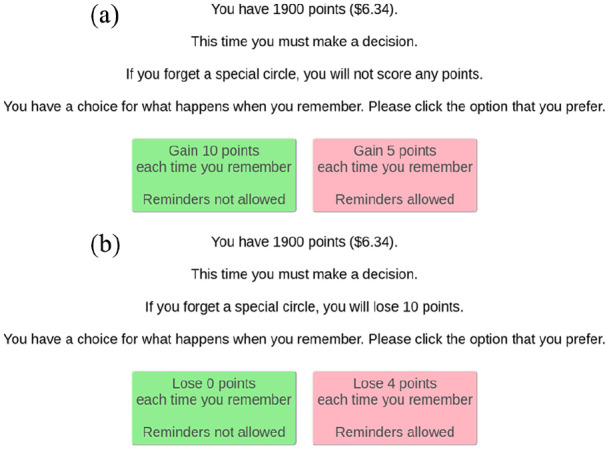
Example instructions for the free-choice trials. Before beginning a free-choice trial, participants were given the options to use their internal memory or reminders. (a) In the gain condition, if participants selected to use their internal memory, they would score 10 points per target circle remembered. However, if participants chose to use reminders, they would earn a smaller number of points per target circle remembered (2–8). (b) In the loss condition, participants were given points at the beginning of the block and lost either 0 points per target circle remembered if they used internal memory or a greater number of points (2–8) if they chose to use reminders. Every trial (forced internal, forced external, free choice) across both conditions constituted a 25 circle sequence, of which 10 were target circles. In terms of reward, the two conditions are mathematically equivalent.

The main experiment was split into two counterbalanced blocks: gain and loss. In the gain condition, participants chose between receiving 10 points for each remembered target circle or a smaller number of points (2–8) to use reminders (Figure 3a), as described above. This matches the version of the task used in previous experiments (Gilbert et al., 2020), and was the focus of analyses for the present study. The loss condition (Figure 3b) was included so that it could be compared with the gain condition as part of a separate project (https://osf.io/8zvf6/), which will be reported in a separate article. During the loss condition, participants received points before the beginning of the block. They were then presented with the choice between (1) using their own memory and keeping all their points (losing 0) each time they correctly remembered target circles, or (2) using reminders, and losing points every time they remembered (2–8). In terms of reward, the two conditions are mathematically equivalent.

For a demonstration, the entire experiment can be accessed here: http://ucl.ac.uk/sam-gilbert/demos/CWPK1/start.html

In addition, the full source code to run the experiment, including all implementation details and instructions, has been uploaded to OSF (https://osf.io/sm3tw/).

#### Apparatus

Participants completed the task via their computer’s web browser. Participation was only to be permitted if the browser window had dimensions of at least 500 × 500 pixels. The square box containing the circles was sized at 80% of the horizontal or vertical extent of the browser window, whichever was smaller. Each circle had a radius of 5.5% of the width/height of the box, and all circles were initially placed so that they fall within a central portion of the box with dimensions sized at 56% of the total width/height, so that no circles were adjacent to any of the edges of the box at the beginning of the trial.

#### Procedure

1. Informed consent.
2. Practice trials and metacognitive judgements.
3. Experimental instructions for gain or loss condition (randomised).
4. Experimental Block 1 (gain or loss). Participants performed a total of 13 trials. On odd-numbered trials, participants were given a free choice between using internal memory (10 points per target circle) or reminders (2–8 points per target circle, presented in random order). On even-numbered trials, participants alternated between the forced-external and forced-internal trials, with the starting trial type (external or internal) randomised between participants, and counterbalanced between gain/loss conditions.
5. Experimental instructions for the other condition (gain or loss).
6. Experimental Block 2 (gain or loss; 13 trials as above).
7. Questionnaires (fixed order: STAI, PSWQ).

#### Reward

Participants were told that they were scoring points, where 300 points was equivalent to $1. They received 600 points at the beginning of the experiment. Then they were able to earn (or keep) up to 1,300 points (i.e., 100 points per trial) in each half of the experimental trials. Therefore, the earnings could range between 600 points ($2) and 3,200 points ($10.67). The experiment was advertised as having a base payment of $2, which participants received simply for taking part, with the additional earnings sent to participants afterwards as a bonus payment.

#### Analysis

##### Dependent measures

- Forced-internal accuracy (ACC_FI_). This is the mean target accuracy (proportion of target circles correctly dragged to the instructed location) on forced-internal trials.
- Forced-external accuracy (ACC_FE_). This is the mean target accuracy (proportion of target circles correctly dragged to the instructed location) on forced-external trials.
- Optimal indifference point (OIP). This is the value for target circles offered with reminders at which an unbiased individual should be indifferent between the two options, based on the accuracy in the forced-internal and forced-external trials (ACC_FI_ and ACC_FE_). As in Gilbert et al. (2020), this was calculated as

$$\mathrm{OIP}\;=\;(10\times {\mathrm{ACC}}_{\mathrm{FI}})/{\mathrm{ACC}}_{\mathrm{FE}}$$

If the OIP was less than 2 or greater than 8, it was set to the relevant lower or upper bound. This was so that the potential values of the OIP would match the potential values of the point at which they were actually indifferent, which was bound by their choices for values 2 to 8.

- Actual indifference point (AIP). This is the estimated point at which participants were actually indifferent to the two strategy options. As in Gilbert et al. (2020), this was calculated by fitting a sigmoid curve to the strategy choices (0 = own memory; 1 = reminders) across the seven target values (2–8), using the R package “quickpsy” (Linares & López-Moliner, 2016) bounded to the range 2 to 8.
- Reminder bias. This is defined as OIP – AIP, which will yield a positive value for a participant biased towards using more reminders than would be optimal, and a negative value for a participant biased towards using fewer reminders than would be optimal.
- Internal metacognitive bias. This is the difference between metacognitive confidence and actual accuracy on forced-internal trials. A positive number would indicate overconfidence of their own memory abilities.
- External metacognitive bias. This is the difference between metacognitive confidence and actual accuracy on forced-external trials. A positive number would indicate overconfidence of their performance when using reminders.

Each of the previous seven measures was calculated separately for the gain and loss conditions.

- Internal metacognitive confidence. This is the response made to the metacognitive accuracy prediction following practice trials using internal memory (see Figure 1).
- External metacognitive confidence. This is the response made to the metacognitive accuracy prediction following practice trials using reminders.

##### Exclusion criteria

Participants were excluded if (1) their accuracy in the forced-internal condition was lower than 10%, averaged across the gain and loss conditions; (2) accuracy in the forced-external condition was lower than 70%, averaged across the gain and loss conditions; (3) accuracy on the forced-internal trials was higher than forced-external trials in either condition; (4) there was a negative point biserial correlation between points offered for correct responses on each trial using reminders (2–8) and choice of strategy (0 = own memory, 1 = reminders; this excludes participants who were more likely to set reminders when it earned them fewer points, suggesting random strategy selection); (5) reminder bias score (averaged across the gain and loss conditions) exceeded 3 median absolute deviation units (MAD; Leys et al., 2013); (6) difference in reminder bias scores between the two conditions exceeded 3 MAD units; and (7) internal metacognitive bias score exceeded 3 MAD units. Data collection continued until the study had the appropriate power (*N* = 300) following exclusion (64 excluded).

##### Statistical tests

All Frequentist analyses were run in RStudio (RStudio Team, 2019). These constituted a series of two-tailed paired-sample *t* tests, one-sample *t* tests, Pearson’s correlations, and linear regressions (enter method). As described in the original pre-registration, our analyses were predominantly restricted to the gain condition only, as this condition more closely replicated the procedure used in previous studies. A comprehensive analysis of the loss condition will be reported separately (see https://osf.io/8zvf6/). We have also supplemented our analyses by providing their Bayesian equivalents in JASP (JASP Team, 2019). Here, we used JASP’s default priors: Bayesian paired-sample *t* test (Cauchy scale = .707); Bayesian one-sample *t* test (Cauchy scale = .707); Bayesian correlation pairs (stretched beta prior width = 1); and Bayesian linear regression (Jeffreys-Zellner-Siow *r* scale = .354; beta binomial *a* = 1, *b* = 1). All Bayes Factors are reported as BF_10_ and winning models in the linear regressions were defined as those with the highest BF_10_ relative to the null (intercept only model). The relative predictability of models in the linear regressions was calculated by dividing BF_10_ between models. To aid in interpretation of Bayes factors, we have used commonly adopted semantic labels for Bayes Factors when describing our findings (anecdotal [1–3], substantial [3–10], strong [10–30], very strong [30–100], decisive [>100]; Jeffreys, 1998).

## Results

### Background analyses

**Figure 4.**
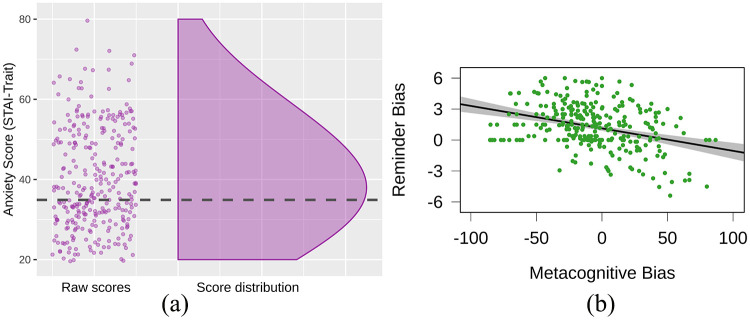
(a) Distribution of trait anxiety scores. Dashed line refers to US “Working Adult” average (Spielberger, 1983). Plotted using RainCloudPlots (Allen et al., 2019). (b) Correlation coefficient slope with 95% confidence interval between metacognitive bias and reminder bias. Positive reminder bias scores indicate overuse of reminders and negative metacognitive bias scores indicate underconfidence of memory.

The following analyses were performed to characterise the basic performance of the experimental task and check whether previous findings were replicated. These analyses do not test any particular hypotheses for the present study but provide further information that may be useful for interpretation of the hypothesis-testing analyses. We have also provided descriptive plots for the distribution of anxiety scores (Figure 4a) and metacognitive/reminder measures ((Supplementary Material 6).

We characterised basic performance of the task by comparing accuracy between the forced-internal and forced-external conditions. Here, we saw decisive evidence for a difference between the two conditions, *t*(332.03) = –25.3, *p* < .0001, BF_10_ > 100, BF_01_ < .01, wherein accuracy was lower for the forced-internal (*M* = 64.89%, *SD* = 21.80) versus forced-external condition (*M* = 97.60%, *SD* = 5.13). We then tested for internal metacognitive bias (defined as predicted internal accuracy minus actual accuracy in the forced-internal condition). We saw very strong evidence that internal metacognitive bias was below 0, *M* = –6.91, *t*(299) = –3.62, *p* < .001, BF_10_ = 36.78, BF_01_ = 0.03, indicating that participants were underconfident in their own memory abilities. We also conducted an analogous analysis on the external metacognitive bias score (i.e., predicted accuracy with reminders minus actual accuracy in the forced-external condition). There was decisive evidence that external metacognitive bias was below 0, *M* = –11.35, *t*(299) = –12.92, *p* < .0001, BF_10_ > 100, BF_01_ < .01, indicating participants were underconfident in their performance on the task with reminders. We investigated reminder bias scores (defined as OIP minus AIP). There was decisive evidence that reminder bias was greater than 0, *M* = 1.28, *t*(299) = 10.32, *p* < .0001, BF_10_ > 100, BF_01_ < .01, indicating that participants typically used more reminders than was optimal. Finally, we investigated whether internal metacognitive bias was associated with reminder bias, as we have found previously (Gilbert et al., 2020). Replicating previous results, there was decisive evidence that internal metacognitive bias was inversely correlated with reminder bias, *r*(298) = –.34, *p* < .0001, BF_10_ > 100, BF_01_ < .01 (Figure 4b), that is, underconfidence in memory abilities was associated with increased reminder usage.

### Key hypotheses

**Figure 5.**
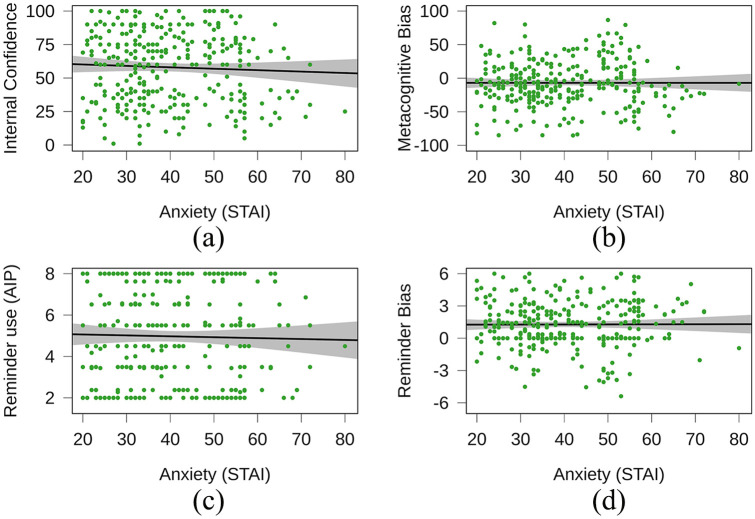
Correlation coefficient slopes with 95% confidence intervals for anxiety and (a) internal confidence, (b) metacognitive bias, (c) reminder usage, and (d) reminder bias. Lower confidence (a) and metacognitive bias (b) scores indicate lower or underconfidence, respectively. Higher reminder use (c) and bias (d) indicate higher or overuse of reminders, respectively.

Our key hypotheses were tested using a series of Pearson’s correlations (Hypotheses 1–5) and a linear regression (enter method; Hypothesis 6). First, we tested whether trait anxiety negatively correlated with participants’ unaided accuracy on our delayed intentions task. We saw substantial evidence that trait anxiety and accuracy in the forced internal were not correlated, *r*(298) = –.06, *p* *=* .*29*, BF_10_ = 0.13, BF_01_ = 7.97. Second, we tested whether trait anxiety would negatively correlate with participants’ confidence in their unaided ability on the task. We saw substantial evidence that there was no correlation between trait anxiety and participants’ confidence in their ability to perform the task, *r*(298) = –.05, *p* = .39, BF_10_ = 0.10, BF_01_ = 9.54 (Figure 5a). Third, we tested whether trait anxiety would negatively correlate with participants’ metacognitive bias for their ability to perform the delayed intentions task. We saw strong evidence that there was no correlation between trait anxiety and internal metacognitive bias, *r*(298) = –.00, *p* = 1.00, BF_10_ = 0.07, BF_01_ = 13.83 (Figure 5b). Fourth, we predicted that trait anxiety would positively correlate with participants’ propensity to use reminders on our delayed intentions task (i.e., AIP). We saw strong evidence to suggest there was no correlation between trait anxiety and participants’ AIP, *r*(298) = –.02, *p* = .67, BF_10_ = 0.08, BF_01_ = 12.65 (Figure 5c). Fifth, we predicted that trait anxiety would positively correlate with participants’ bias towards the use of reminders in the delayed intentions task. We saw strong evidence to suggest there was no correlation between trait anxiety and participants’ reminder bias, *r*(298) = .00, *p* = .94, BF_10_ = 0.07, BF_01_ = 13.80 (Figure 5d).

Finally, we predicted that both anxiety and metacognitive bias would account for unique variance in a model of reminder bias. We performed a linear multiple regression on the reminder bias score, with factors trait anxiety and internal metacognitive bias as predictor variables (enter method). Congruent with the previous analyses, internal metacognitive bias retained predictive power (β = –.02, *p* < .0001) while trait anxiety did not account for unique variance (β = .00, *p* *=* .94). For the Bayesian linear regression, the winning model was that which only included internal metacognitive bias (BF_10_ > 100), which was substantially (7 times) better than the model which included metacognitive bias and trait anxiety (BF_10_ > 100), decisively (>1,000 times) better than the null model (BF_10_ = 1), and decisively (>1,000 times) better than the trait anxiety only model (BF_10_ = 0.13).

### Additional exploratory analyses

We performed additional exploratory analyses to provide further characterisation of our dataset. Given the large number of tests provided, these were considered as exploratory tests rather than key hypothesis-testing analyses, the full extent of which can be found in the (supplemental materials. First, we repeated the above analyses, using the worry measure instead of trait anxiety ((Supplementary Material 1). We found no evidence for a relationship in this domain, nor did we find including both trait anxiety and worry as predictors in a regression model to alter the inference. We also found that there was a positive correlation between PSWQ and STAI (*r* = .77, *p* < .0001, BF_10_ > 100, (Supplementary Material 2), consistent with previous results (*r* = .64, Meyer et al., 1990). This shows that even though our measure of trait anxiety did not correlate with performance of the experimental task, it did relate as expected to our measure of worry. We found no evidence to suggest anxiety or worry correlated with participants’ confidence in their ability to perform the task with reminders, and external metacognitive bias ((Supplementary Material 3). Finally, we performed additional analyses using data from the loss rather than the gain condition. Results were similar: anxiety did not correlate with internal accuracy, *r*(298) = –.05, *p* = .39, BF_01_ = 0.11, BF_10_ = 9.52; internal metacognitive bias, *r*(298) = –.01, *p* = .90, BF_10_ = 0.07, BF_01_ = 13.73; reminder use, *r*(298) = –.05, *p* = .43, BF_10_ = 0.10, BF_01_ = 10.15; or reminder bias, *r*(298) = .03, *p* = .57, BF_10_ = 0.08, BF_01_ = 11.80, in the loss condition ((Supplementary Material 4), providing further evidence for the null.

We have also provided additional, post hoc analyses which may address outstanding questions. As the order in which conditions were presented (gain first vs. loss first) had a significant impact on internal accuracy for the gain condition, and consequently our calculation of internal metacognitive bias, we re-analysed our data separately for each counterbalancing group ((Supplementary Material 8). As STAI scores were not normally distributed, and we did not screen for anxiety disorders, we provide group comparison analyses between participants on the upper and lower quartiles of STAI scores ((Supplementary Material 5), as well as analyses restricted to less anxious participants ((Supplementary Material 10). Finally, we have included a re-analysis of the data on all participants without any exclusions (*N* = 364; (Supplementary Material 9). None of the above analyses provided any evidence towards the alternative hypotheses, and thus did not change our overall inference.

## Discussion

There is a myriad of experimental evidence demonstrating that anxiety leads to altered behaviour and cognitions (Robinson et al., 2013). In particular, prior studies have outlined a relationship between trait anxiety and metacognitive belief (Spada et al., 2010; Wells & Cartwright-Hatton, 2004). Our previous work has established a link between metacognitive belief and use of reminders for future intentions (Boldt & Gilbert, 2019; Gilbert et al., 2020). The present article set to bridge these areas by carrying out correlational analyses between individual differences in trait anxiety, metacognitive confidence, and offloading behaviour on a delayed intentions paradigm. Specifically, we correlated trait anxiety with the following measures on our task: unaided accuracy, metacognitive confidence in one’s ability to perform the task, internal metacognitive bias (the discrepancy between confidence and performance), reminder usage, and reminder bias (optimality of reminder-setting). Despite an implied relationship from the previous literature and a sample size (*N* = 300) powered to detect effects bigger than *r* = .161, our preregistered experiment provided no evidence for any correlation between trait anxiety and the behavioural data on our delayed intentions task.

Our first hypothesis tested whether higher trait anxiety resulted in worse unaided performance on our delayed intentions task. This was motivated by the notion that anxiety can result in impaired memory processes (Shackman et al., 2006). On one hand, no relationship may exist between trait anxiety and memory in the context of a delayed intentions paradigm. Alternatively, it has been suggested that anxiety only impairs performance at low cognitive loads (see Vytal et al., 2012). This may be because anxiety results in disruptive worrying thoughts while at low load, but this could dissipate at higher loads, with attentional resources being re-focussed onto the task at hand. The delayed intentions paradigm we utilised is relatively demanding, and performance was well below ceiling (mean accuracy = 65%). Therefore, detrimental effects of anxiety on performance could have been mitigated by difficulty-driven attention. However, our previous work has demonstrated that cognitive load is not always a sufficient explanation of the impact of anxiety on cognition (Sarigiannidis et al., 2020). Nevertheless, future work might seek to retest whether a correlation exists for our task at lower loads (e.g., fewer target circles).

The second and third hypotheses tested whether trait anxiety correlated with confidence to perform the task, and the accuracy of this confidence (metacognitive bias). These hypotheses were driven by work relating scores on trait anxiety to the MCQ-30 (Spada et al., 2010; Wells & Cartwright-Hatton, 2004). This latter measure includes asking participants to rate general confidence in their memory abilities. However, confidence measures for our task did not appear to hold a relationship with trait anxiety. Despite a prior experiment demonstrating correlations between confidence in our paradigm and the MCQ-30 (Boldt & Gilbert, 2019), it may be that the explanatory variance shared between these two measures does not overlap with trait anxiety. In other words, anxiety may correlate with general feelings of confidence as reported in a questionnaire such as the MCQ-30, but not performance predictions in a specific task. Further experiments attempting to replicate our findings should also include the MCQ-30 measures to grasp both a domain-general measure of metacognitive belief and task-specific confidence measures.

Although we took measures of confidence to perform the task, these do not capture the accuracy of beliefs. As such, we calculated metacognitive bias scores, a metric of the discrepancy between self-reported confidence and accuracy. While participants were generally underconfident in their ability to perform the task, we saw no correlation between trait anxiety and metacognitive bias. We can infer that high-anxiety individuals show similar levels of metacognitive bias as low-anxiety individuals.

An overarching goal of metacognition research is to develop ways to optimise peoples’ behaviour in line with their cognitive abilities (Gilbert et al., 2020). It would therefore be constructive to examine anxiety in such a context. We previously found that providing metacognitive advice can reduce bias in our delayed intentions task (Gilbert et al., 2020). It would be informative to investigate whether anxiety acts as a mediating factor between metacognitive advice and updating of confidence. More generally, does anxiety reduce individuals’ propensity to update cognitive confidence through external advice?

Our fourth, fifth, and sixth hypotheses investigated the extent to which trait anxiety was related to participants’ use of reminders. We posited that a correlation between reminder usage and anxiety could be due to lowered confidence and/or through unique influences external to higher-order cognition. Yet, we did not observe any relationship between anxiety and reminder usage. This suggests there may be no difference between high- and low-anxiety individuals in the frequency and (sub)optimality of reminder-setting. Again, it would be useful for future work to test whether anxiety acts as a barrier (or even facilitator) to the alteration of reminder usage following external advice.

Finally, two caveats should be noted with the present study. First, this work did not explicitly screen for anxiety disorders; rather, we looked across a spectrum of individuals at the population level. Thus, we do not make a distinction between maladaptive versus adaptive anxiety. As such, we have provided additional analyses ((Supplementary Material 5/10) comparing participants on the extremities of the STAI scale, although this did not change our inference. Yet, without explicit screening and population comparisons, we cannot distinguish to what extent our findings may be driven by the presence (or lack) of maladaptive anxiety disorders. Second, our study was inherently correlational in nature and did not explicitly induce state anxiety at a within-subjects level. Understanding that our experiment was completed remotely, probably at home, it is feasible that participants were in relatively low anxiety *states* during the experiment, even if they otherwise held generally high-anxiety *traits*. This is not something that could have easily been addressed with the state section of the STAI ((Supplementary Material 7). Future work could implement within-subjects state anxiety manipulations such as threat-of-shock, anxiety-relevant stimuli, or time pressure paradigms. This would help elucidate whether increases in *state* anxiety lead to increased metacognitive and reminder bias, despite the lack of a relationship between the latter measures and *trait* anxiety.

## Conclusion

Our present study bridged the gap between two different areas of research, namely, affective processes and metacognition. Previous findings suggested a relationship may exist between trait anxiety, metacognitive bias, and reminder bias. Specifically, prior work implied higher trait anxiety results in a general underconfidence in cognitive abilities, and possibly excessive reminder usage. Within the context of a delayed intentions task, we found evidence against a relationship between trait anxiety and memory abilities, confidence, or reminder usage. Highly anxious individuals were similar in their optimality of reminder-setting as low-anxiety individuals. Future work may seek to expand our findings by repeating the task across different cognitive loads, using clinical comparisons, and manipulating anxiety in-lab.

## Supplemental Material

QJE-STD-20-191.R1-Supplementary_Material – Supplemental material for Trait anxiety does not correlate with metacognitive confidence or reminder usage in a delayed intentions task Click here for additional data file. Supplemental material, QJE-STD-20-191.R1-Supplementary_Material for Trait anxiety does not correlate with metacognitive confidence or reminder usage in a delayed intentions task by Peter A Kirk, Oliver J Robinson and Sam J Gilbert in Quarterly Journal of Experimental Psychology
