# Supplementary material for: Trait anxiety does not correlate with metacognitive confidence or reminder usage in a delayed intentions task
Source: Q J Exp Psychol (Hove). 2020 Nov 12;74(4):634–44. doi: 10.1177/1747021820970156 (PMC8044609; doi:10.1177/1747021820970156)
Supplement: QJE-STD-20-191.R1-Supplementary_Material – Supplemental material for Trait anxiety does not correlate with metacognitive confidence or reminder usage in a delayed intentions task [file QJE-STD-20-191.R1-Supplementary_Material.docx]

**Supplementary Materials**

**Trait anxiety does not correlate with metacognitive confidence or reminder usage in a delayed intentions task**

**by Peter A. Kirk., Oliver J. Robinson, & Sam J. Gilbert, 2020**

**Supplement 1. Correlational analyses between individual differences in worry and behavioural data**.

We tested whether worry negatively correlated with participants’ unaided accuracy on our delayed intentions task. We saw strong evidence that worry and accuracy in the forced internal were not correlated (*r*(298) = .01, *p* = .83, BF_10_ = 0.07, BF_01_ = 13.50). Second, we tested whether worry would negatively correlate with participants confidence in their unaided ability on the task. We saw strong evidence that there was no correlation between worry and participants’ confidence in their ability to perform the task (*r*(298) = -.01, *p* = .80, BF_10_ = 0.07, BF_01_ = 13.39). Third, we tested whether worry would negatively correlate with participants metacognitive bias for their ability to perform the delayed intentions task. We saw strong evidence to suggest there was no correlation between worry and internal metacognitive bias (*r*(298) = -.02, *p* = .73, BF_10_ = 0.08, BF_01_ = 13.01). Fourth, we predicted that worry would positively correlate with participants’ propensity to use reminders in our delayed intentions task (i.e. the Actual Indifference Point, AIP). We saw strong evidence to suggest there was no correlation between worry and participants’ AIP (*r*(298) = .03, *p* = .57, BF_10_ = 0.08, BF_01_ = 11.82). Fifth, we predicted that worry would positively correlate with participants’ bias toward the use of reminders in a delayed intentions task. We saw strong evidence to suggest there was no correlation between worry and participants’ reminder bias (*r*(298) = .00, *p* = .99, BF_10_ = 0.07, BF_01_ = 13.83). Lastly, we predicted that both worry, and metacognitive bias would account for unique variance in a model of reminder bias. We performed a linear multiple regression on the reminder bias score, with factors worry and internal metacognitive bias inputted using the enter method. Congruent with the previous analyses, internal metacognitive bias retained predictive power (β = -.02, *p* < .0001), while worry did not account for unique variance (β = .00, *p =* .91). For the Bayesian linear regression, the winning model was that which only included internal metacognitive bias (BF_10_ > 100, , BF_01_ < .01), which was substantially (7 times) better than the model which included metacognitive bias and worry (BF_10_ > 100, BF_01_ < .01), decisively (>1000 times) better than the null model (BF_10_ = 1, BF_01_ = 1), and decisively (>1000 times) better than the worry only model (BF_10_ = 0.13, BF_01_ = 7.88). We also performed a linear multiple regression analysis of the reminder bias score with factors anxiety, worry, and internal metacognitive bias. This tested whether all three factors could account for unique variance in the reminder bias. Congruent with the previous analyses, internal metacognitive bias retained predictive power (β = -.02, *p* < .0001), while neither anxiety (β = .00, *p =* .80) nor worry accounted for unique variance (β = .00, *p =* .79). For the Bayesian linear regression, the winning model was that which only included internal metacognitive bias (BF_10_ > 100, BF_01_ < .01), which was: substantially (7 times) better than the models including internal metacognitive bias + worry (BF_10_ > 100, BF_01_ < .01) and internal metacognitive bias + anxiety (BF_10_ > 100, BF_01_ < .01); and decisively (>1000 times) better than the internal metacognitive bias + anxiety + worry model (BF_10_ > 100, BF_01_ < .01, null model (BF_10_ = 1, BF_01_ < .01), anxiety only model (BF_10_ = 0.13, BF_01_ = 7.86), worry only model (BF_10_ = 0.13, BF_01_ = 7.88), and anxiety + worry model (BF_10_ = 0.02, BF_01_ = 40.28).

**Supplement 2. Correlation between individual differences in trait anxiety and worry.**

We conducted an analysis to determine the extent to which our trait anxiety and worry measure were correlated. Our analysis provided decisive evidence that these two measures were highly correlated (*r*(298) = .77, *p* < .0001, BF_10_ > 100, BF_01_ < .01).

**Supplement 3. Correlating anxiety and worry to external confidence.**

We investigated whether anxiety or worry were correlated with participants’ confidence in their ability to perform the task with reminders. We found substantial evidence for no correlation between trait anxiety and external confidence (*r*(298) = -.06, *p* = .27, BF_10_ = 0.13, BF_01_ = 7.54), and strong evidence for no correlation between worry and external confidence (*r*(298) = -.03, *p* = .64, BF_10_ = 0.08, BF_01_ = 12.42). Next, we investigated whether anxiety or worry were correlated with participants’ external metacognitive bias (i.e. predicted accuracy with reminders minus accuracy in the forced external condition). We found strong evidence for no correlation between anxiety and external metacognitive bias (*r*(298) = -.00, *p* = .93, BF_10_ = 0.07, BF_01_ = 13.78), and strong evidence for no correlation between worry and external metacognitive bias (*r*(298) = -.00, *p* = .97, BF_10_ = 0.07, BF_01_ = 13.82).

**Supplement 4. Analysis of loss condition.**

Although all previous analyses were based on data from the gain condition only, we also conducted analyses using the data from the loss condition, to investigate whether any findings related to anxiety or worry were found with this version of the task. In particular, we tested our key hypotheses using data from the loss condition and between conditions, along with analogous analyses using the worry measure instead of anxiety. Internal confidence was not included, as this did not differ between conditions.

Regarding trait anxiety and measures in the loss condition, we found substantial evidence for no correlation between anxiety and internal accuracy (*r*(298) = -.05, *p* = .39, BF_10_ = 0.11, BF_01_ = 9.52), and strong evidence for no correlations with internal metacognitive bias (*r*(298) = -.01, *p* = .90, BF_10_ = 0.07, BF_01_ = 13.73), AIP (*r*(298) = -.05, *p* = .43, BF_10_ = 0.10, BF_01_ = 10.15), and reminder bias (*r*(298) = .03, *p* = .57, BF_10_ = 0.08, BF_01_ = 11.80). Regarding worry and metacognitive/reminder measures in the loss condition, we found strong evidence for no correlations between worry and internal accuracy (*r*(298) = .00, *p* = .96, BF_10_ = 0.07, BF_01_ = 13.82), internal metacognitive bias (*r*(298) = -.01, *p* = .81, BF_10_ = 0.07, BF_01_ = 13.46), AIP (*r*(298) = -.02, *p* = .68, BF_10_ = 0.08, BF_01_ = 12.68), and reminder bias (*r*(298) = .05, *p* = .42, BF_10_ = 0.10, BF_01_ = 10.07)

We next conducted multiple regressions on the reminder bias data in the loss condition. Firstly, we built a linear regression using reminder bias (loss) as the dependent variable and internal metacognitive bias (loss) and trait anxiety as predictor variables. Congruent with the gain condition, internal metacognitive bias retained predictive power (β = -.02, *p* < .0001), while anxiety did not account for unique variance (β = .01, *p* =.58). For the Bayesian linear regression, the winning model was that which only included internal metacognitive bias (BF_10_ > 100, BF_01_ < .01), which was substantially (6 times) better than the model which included metacognitive bias and anxiety (BF_10_ > 100, BF_01_ < .01), decisively (>1000 times) better than the null model (BF_10_ = 1, BF_01_ = 1), and decisively (>1000 times) better than the anxiety only model (BF_10_ = 0.15, BF_01_ = 6.76). Secondly, we built a linear regression using reminder bias (loss) as the dependent variable and used internal metacognitive bias and worry as predictor variables. Congruent with the gain condition, internal metacognitive bias retained predictive power (β = -.02, *p* < .0001), while worry did not account for unique variance (β = .01, *p* =.45). For the Bayesian linear regression, the winning model was that which only included internal metacognitive bias (BF_10_ > 100, BF_01_ < .01), which was substantially (5 times) better than the model which included metacognitive bias and worry (BF_10_ > 100, BF_01_ < .01), decisively (>1000 times) better than the null model (BF_10_ = 1, BF_01_ = 1), and decisively (>1000 times) better than the worry only model (BF_10_ = 0.17, BF_01_ = 5.80).

Lastly, we conducted correlational analyses on individual differences in anxiety/worry, and within-subject discrepancies in reminder bias (reminder bias(loss) – reminder bias(gain)). Here, we found strong evidence for no correlation between reminder bias (loss-gain) and anxiety (*r*(298) = .03, *p* = .64, BF_10_ = 0.08, BF_01_ = 12.37), and strong evidence for no correlation between reminder bias (loss-gain) and worry (*r*(298) = .04, *p* = .45, BF_10_ = 0.10, BF_01_ = 10.48).

**Supplement 5. Between-groups analysis of lower/upper anxiety quartiles.**

In addition to our correlational analyses, we conducted further testing to look at participants on the extremities of the STAI scale. Here, we split participants into two groups (lower quartile vs upper quartile STAI scores), and repeated frequentist and Bayesian t-test equivalents on key hypotheses (H1-H5). These groups did not differ in accuracy (t(150.96) = 1.00, p = .32, BF_10_ = .28, BF_01_ = 3.62), confidence (t(151.64) = 0.22, p = .83, BF_10_ = .18, BF_01_ = 5.63), metacognitive bias (t(150.21) = -0.48, p = .63, BF_10_ = .19, BF_01_ = 5.18), reminder use (t(151.80) = 0.61, p = .54, BF_10_ = .21, BF_01_ = 4.85), or reminder bias (t(149.17) = -0.27, p = .78, BF_10_ = .18, BF_01_ = 5.56). This further supports our inference that trait anxiety does not impact metacognitive confidence or reminder usage as measured in our study.

**Supplement 6. Descriptive plots for the distribution of metacognitive bias/reminder measures.**


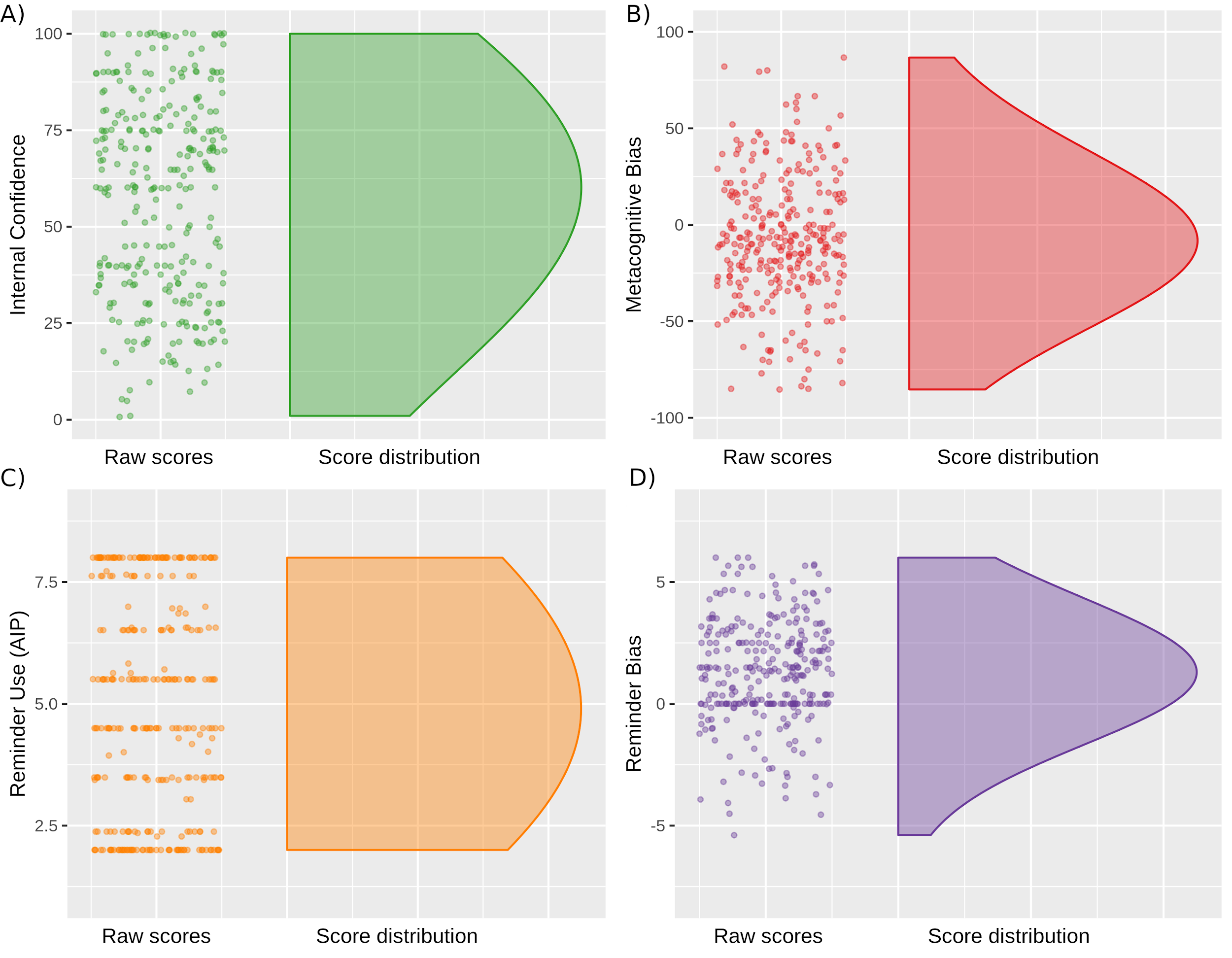


**Supplement 7. Correlation between the state and trait sections of the STAI.**

A reason for only selecting the trait section of the STAI scale was due to our previous observations that the trait and state sections correlate highly. As such, we demonstrate a re-analysis of a dataset in our group from an online experiment (also conducted on MTurk) which used both state and trait measures (N = 1060; Daniel-Watanabe et al., 2020). This demonstrates a very high correlation between these measures (*r*(1058) = .83, *p* < .0001, BF_10_ > 1000). As such, we believe the best way to assess influences of state level anxiety would be through in-lab anxiety inductions, not through STAI-measured state anxiety.


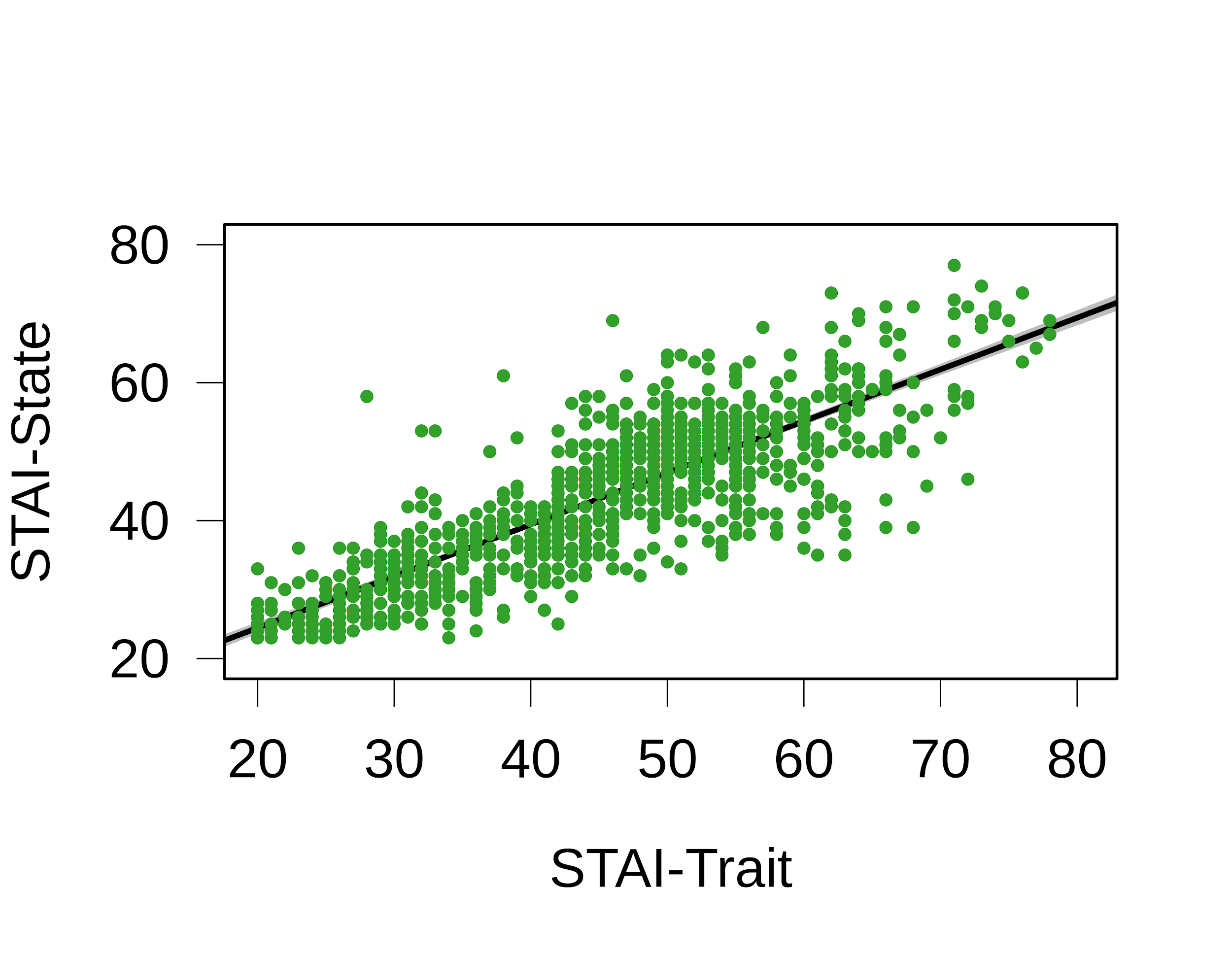


Reference

Daniel-Watanabe, L., McLaughlin, M., Gormley, S., & Robinson, O. J. (2020). Association Between a Directly Translated Cognitive Measure of Negative Bias and Self-reported Psychiatric Symptoms. *Biological Psychiatry: Cognitive Neuroscience and Neuroimaging*. <https://doi.org/10.1016/j.bpsc.2020.02.010>.

**Supplement 8. Effects of block order.**

To investigate practice effects, we split participants by counterbalancing group (GainFirst = gain condition first, GainSecond = gain condition second) and re-analysed data. Indeed, there was decisive evidence for a main effect of block order, such that GainSecond participants performed better on forced-internal trials in the gain condition (M = 69.93%, SD = 22.31) than GainFirst participants (M = 60.17%, SD = 20.26; t(290.32) = -3.96, p < .0001, BF10 > 100). Consequently, there was substantial evidence to suggest a difference in metacognitive bias between these groups (mean difference = 11.18%, t(279.51) = 2.94, p = .004, BF10 = 8.02). To ensure that our findings were not driven by any of these effects, we reanalyzed our hypotheses separately for each group. Despite the difference in accuracy and metacognitive bias, this did not change our inference.

In the GainFirst group there were no correlations between anxiety and: internal accuracy (r(143) = -.03, p = .71, BF10 = .11, BF01 = 9.00), confidence (r(143) = -.02, p = .81, BF10 = .11, BF01 = 9.35), metacognitive bias (r(143) = .00, p = 1.00, BF10 = .10, BF01 = 9.62), reminder use (r(143) = -.01, p =.88, BF10 = .10, BF01 = 9.52), or reminder bias (r(153) = -.00, p =.96, BF10 = .10, BF01 = 9.61). Similarly, in the GainSecond group, there were no correlations between anxiety and: internal accuracy (r(153) = -.09, p = .22, BF10 = .21, BF01 = 4.76), confidence (r(153) = -.07, p = .34, BF10 = .16, BF01 = 6.30), metacognitive bias (r(153) = .00, p = 1.00, BF10 = .10, BF01 = 9.95), reminder use (r(153) = -.04, p =.63, BF10 = .11, BF01 = 8.90), or reminder bias (r(153) = -.01, p =.89, BF10 = .10, BF01 = 9.86).

**Supplement 9. Re-analysis of data without exclusion criteria.**

After re-analysing the data without any exclusion criteria applied (N= 364), anxiety still did not demonstrate a correlation with internal accuracy (*r*(362) = -.09, *p* = .08, BF_10_ = .29, BF_01_ = 3.39), confidence (*r*(362) = -.02, *p* = .65, , BF_10_ = .07, BF_01_ = 13.71), metacognitive bias (*r*(362) = .04, *p =* .42, BF_10_ = .09, BF_01_ = 11.07), reminder use (*r*(362) = -.03, *p =*.55, BF_10_ = .08, BF_01_ = 12.75), and reminder bias (*r*(362) = -.01, *p =*.86, BF_10_ = .07, BF_01_ = 15.01).

**Supplement 10. Median split re-analysis of data.**

To rule out the possibility that results are being masked by participants with potential maladaptive anxiety disorders, we ran correlations between anxiety scores and metacognitive/reminder variables for participants with STAI scores less than or equal to our sample median (37.5; N = 150). Here, anxiety did not correlate with accuracy (*r* = .03, *p* = .70, BF_10_ = 0.11, BF_01_ = 9.10), confidence (*r* = .04, *p* = .62, BF_10_ = 0.12, BF_01_ = 8.66), metacognitive bias (*r* = .01, *p* = .88, BF_10_ = 0.10, BF_01_ = 9.69), reminder use (*r* = .13, *p* = .10, BF_10_ = 0.38, BF_01_ = 2.66), or reminder bias (*r* = -.01, *p* = .23, BF_10_ = 0.21, BF_01_ = 4.80).
